# Transmission of multidrug-resistant tuberculosis within family households by DTM-PCR and MIRU-VNTR genotyping

**DOI:** 10.1186/s12879-022-07188-7

**Published:** 2022-02-26

**Authors:** Jun Chen, Lifeng Chen, Meng Zhou, Gang Wu, Fenglian Yi, Chen Jiang, Qionghong Duan, Meilan Zhou

**Affiliations:** 1 Department of Laboratory Medicine, Wuhan Institute for Tuberculosis Control, Wuhan Pulmonary Hospital, Wuhan, 430030 China; 2 Department of Internal Medicine, Wuhan Institute for Tuberculosis Control, Wuhan Pulmonary Hospital, Wuhan, 430030 China; 3 Department of Tuberculosis Control, Wuhan Institute for Tuberculosis Control, Wuhan Pulmonary Hospital, Wuhan, 430030 China; 4 Pharmaceutical Sciences, School of Pharmacy, University of Pittsburgh, Pittsburgh, USA

**Keywords:** *Mycobacterium tuberculosis*, Multidrug-resistant tuberculosis, Family household, Transmission, MIRU-VNTR genotyping

## Abstract

**Background:**

Drug-resistant tuberculosis (TB) continues to be a public health threat. There are few studies on transmission and genotyping of MDR-TB family households in China. This study aimed to investigate transmission of multidrug-resistant tuberculosis (MDR-TB) within family households by deletion-targeted multiplex polymerase chain reaction (DTM-PCR), mycobacterial interspersed repetitive unit variable number tandem repeats (MIRU-VNTR) genotyping.

**Methods:**

Among 993 MDR-TB patients registered from Wuhan Institute for Tuberculosis Control, drug resistance and the time interval between the index patients and secondary patients were analyzed in 49 MDR-TB patients from 23 families, in which 22 MDR-TB strains from 11 families who had matched strains were genotyped by DTM-PCR and standard 24-loci MIRU-VNTR genotyping method.

**Results:**

The time interval between the index patients and the secondary patients ranged from half a month to 110 months. Thirteen secondary patients developed active MDR-TB within two years and accounted for 50% (13/26) of all secondary patients. Among eleven pairs of MDR-TB families, six pairs had identical genotypes, the cluster rate was 54.5% (12/22); three pairs had a single MIRU-VNTR locus variation. If a single MIRU-VNTR locus variation was tolerated in the cluster definition, the cluster rate raised to 81.8% (18/22).

**Conclusions:**

The family households of MDR-TB patients are at risk for infection of MDR-TB. To reduce transmission, MDR-TB patients should be diagnosed earlier and promptly treated in an effective manner, meanwhile, the close family contacts should be screened for TB infection.

## Background

Drug-resistant tuberculosis (TB) continues to be a public health threat. Worldwide in 2019, close to half a million people developed rifampicin-resistant TB (RR-TB), of which 78% had multidrug-resistant TB (MDR-TB). China ranked the second (14%) among the countries with the largest share of the global burden of MDR-TB [1]. MDR-TB is transmitted through air droplets like drug-susceptible tuberculosis. The high-risk groups most vulnerable to the spread of MDR-TB are close family contacts. Data show that MDR-TB close contacts are often multidrug-resistant when they develop active TB [2, 3]. Among immunocompromised people, the highest risk of developing TB within the first two years after exposure is about 5%-10%, after which the risk is significantly reduced. In the areas with high burden of TB, above half of family members are infected with the same bacterial strain [4]. The WHO “Guidelines for the programmatic management of drug-resistant tuberculosis” recommends that the investigation of MDR-TB contacts should be given priority; close contacts of MDR-TB should be followed up for at least 2 years.

Genotyping of *Mycobacterium tuberculosis* (MTB) strains is important to effectively guide outbreak investigations, define transmission dynamics and assist global epidemiological surveillance of the disease [5]. Owing to its shorter turnaround and simple numerical nomenclature system, mycobacterial interspersed repetitive unit variable number tandem repeats (MIRU-VNTR) genotyping, which is based on 24 standardized loci has replaced IS6110 DNA fingerprinting over the last decade as a gold standard among classical strain typing methods for many applications [5]. In addition, the deletion-targeted multiplex polymerase chain reaction (DTM-PCR), which is also faster and easier to perform, has been considered as a good alternative method to Spoligotyping to identify *Mycobacterium tuberculosis* Beijing strains. The Beijing genotype is predominant among MDR-TB strain in China [6, 7].

Literature analysis revealed studies on transmission and genotyping of MDR-TB family close contacts in China is scarce and limited with case reports [8, 9]. In the current study, we collected data on 993 MDR-TB patients from Wuhan Institute for Tuberculosis Control, a site for National MDR Tuberculosis Prevent and Control Management Demonstration Zone under MDR-TB Prevention and Control Project between January 1, 2007 and March 31, 2018. forty-nine MDR-TB family members were finally chosen for analysis wherein, their drug resistance patterns, and transmission was analysed using DTM-PCR and 24-loci MIRU-VNTR genotyping methods.

## Methods

### Ethics statement

The study was approved by the Medicine Ethics Committee of Wuhan Pulmonary Hospital. All the patients provided informed consent before participation in this study. Ethics in accordance with the Helsinki Declaration on the participation of human subjects in medical research were respected.

### Patients

From January 1, 2007 to March 31, 2018, 993 MDR-TB patients were enrolled in the Wuhan MDR-TB Prevention and Control Project, among them 49 MDR-TB family members representing 23 families (There were two cases in 20 families, and there three cases in three families) were included for analysis in this study.

### Diagnosis of MDR-TB

Sputum specimens were decontaminated with a 2–fourfold volume of 4% NaOH, liquefied at room temperature for 20 min. The processed specimens were inoculated on to Lowenstein-Jensen (L-J) based solid medium for isolation of Mycobacteria. Drug susceptibility testing was performed by using the proportion method on Lowenstein-Jensen (L-J) medium. The following drug critical concentrations were used: isoniazid (INH): 0.2 μg/mL, rifampicin (RFP): 40.0 μg/mL, streptomycin (SM): 4.0 μg/mL, ethambutol (EMB): 2.0 μg/mL, ofloxacin (OFX): 2.0 μg/mL, kanamycin (KM): 20.0 μg/mL. MDR-TB is defined as resistance to at least isoniazid and rifampicin in vitro[10].

### Discovery of a secondary case

MDR-TB patient diagnosis and treatment information was inputted into the China Disease Prevention and Control Information System Tuberculosis Special Report System. Wuhan Institute for Tuberculosis Control set up a MDR-TB clinic, which assigned one doctor and one nurse to be responsible for establishing the medical record information of MDR-TB patients. During the two years of treatment and management, they conducted interviews and screenings for close contacts in MDR-TB families. The case information of close contact in MDR-TB families was collected, such as whether there were cough, fever, night sweat and other symptoms. Sputum specimens from patients with cough, strong TST positive and abnormal chest X-rays were collected for smears and culture.

### Relevant definitions

*Index case* The first MDR-TB patient diagnosed in the family, according to the date of collection of the first culture -positive specimen.

*Secondary case* The MDR-TB patient diagnosed subsequently in the same family.

*MDR-TB close family contact* People living in the same family, or people who spend several hours a day with the MDR-TB patient in the same room.

### The DTM-PCR typing method

Genomic DNA of the isolate was extracted according to the bacterial genomic DNA extraction kit provided by Daan Gene Biology Co., Ltd.

The method reported from Khosravi et al. [11] was used for DTM-PCR typing of all strains. W-Beijing family strains generally lack the RD105 region during the evolution process, while non-Beijing family strains still retain this region. Therefore, W-Beijing family strains can be identified by detecting the deletion of RD105.

### MIRU-VNTR genotyping

MIRU-VNTR genotyping based on standard 24 loci [12] was performed to determine genetic relationships among isolates in this study. The 24 loci include MIRU02, Mtub04, ETRC, MIRU04, MIRU40, MIRU10, MIRU16, Mtub21, MIRU20, QUB11b, ETRA, Mtub29, Mtub30, ETRB, MIRU23, MIRU24, MIRU26, MIRU27, Mtub34, MIRU31, Mtub39, QUB26, QUB4156, and MIRU39. The primers were synthesized by Sangon Biotech (Shanghai) Co., Ltd. Each locus amplification reaction was performed in a total volume of 20 μl consisting of 10 μl 2 × Taq PCR MasterMix (CWBIO), 2 μl 2 μmol/L each primer, 1 μl DNA lysate and 5 μl ddH_2_O. The PCR products were analyzed in a 1.5 ~ 2% agarose gel using a 100 bp DNA ladder as the molecular weight standard. Positive and negative controls were included in each PCR reaction, as H37Rv and H_2_O, respectively. The number of tandem repeats was calculated based on the length of the repeat and flank sequences for each locus. The 24-digit profiles were compared using https://www.miru-vntrplus.org. The unweighted pair-group method with arithmetic mean (UPGMA) was used to construct the MIRU minimum tree.

### Statistical processing

Excel 2013 software was used to manage data. SPSS21.0 (SPSS Inc., Chicago, IL, USA) was used for statistical analysis. The demographic and clinical features of patients were assessed by univariate descriptive analysis.

## Results

### Inclusion and demographics of study subjects

**Fig. 1:**
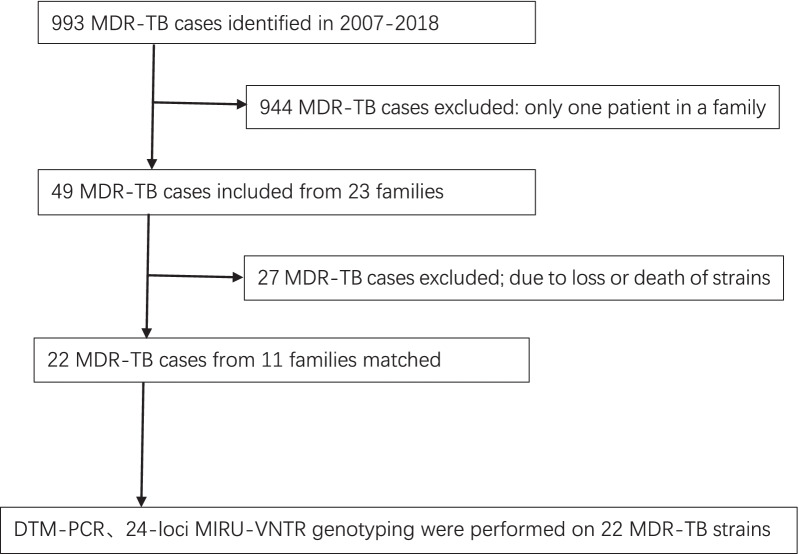
Flow diagram of enrollment to the study

From January 1, 2007 to March 31, 2018, there were 993 MDR-TB patients enrolled in the Wuhan MDR-TB Prevention and Control Project, of which 49 were MDR-TB family members, and they were distributed in 23 families. Forty-nine MDR-TB cases from twenty-three families were included in our study. Twenty-two matched strains from eleven families were typed on DTM-PCR and MIRU-VNTR genotyping (Fig. 1).

Among the 49 MDR-TB patients, there were twenty-nine males and twenty females. Their age ranged from 15 to 72 years old. They were distributed in 23 families, and 40 patients came from 20 families which had two cases per family (one index case and one secondary case); nine patients came from three families, which had three cases per family (one index case and two secondary cases); There were 23 index cases and 26 secondary cases. Among the 26 secondary family cases, there were 15 males, accounting for 57.7%; 11 females, accounting for 42.3%; Their ages ranged from 15 to 68 years old. Among twenty-six secondary cases, six secondary cases had diabetes mellitus type 2 (T2DM) (Table 1).

**Table 1:** Characteristics and drug resistance of index cases and secondary cases

Family number	Gender^a^	Age	Relationship^b^	Comorbidities^c^	Attack interval(month)	Drug-resistance type^d^
A1	M	72	Father*	None	0	INH/RFP/EMB/SM/OFX
	M	40	Son	T2DM	0.5	INH/RFP/EMB/SM/OFX
	M	62	Father*	None	0	INH/RFP
A2	M	35	Son	None	3	INH/RFP/SM
	F	31	Daughter-in-law	None	8	INH/RFP
A3	F	50	Wife*	None	0	INH/RFP/EMB/SM
	M	50	Husband	None	9	INH/RFP/SM/OFX
A4	F	38	Niece*	None	0	INH/RFP/SM
	M	56	Uncle	None	9	INH/RFP
A5	F	22	Daughter-in-law*	None	0	INH/RFP/SM
	M	55	Father-in-law	T2DM	10	INH/RFP/EMB/SM
	M	50	Uncle-in-law	T2DM	46	INH/RFP/SM
A6	F	29	Elder sister*	None	0	INH/RFP/EMB/OFX
	F	26	Younger sister	Chronic renal failure	12	INH/RFP/EMB/OFX
A7	M	52	Younger brother*	None	0	INH/RFP/SM/OFX
	M	57	Elder brother	None	13	INH/RFP/OFX
A8	M	41	Husband*	Hepatitis B virus infection	0	INH/RFP/SM
	F	43	Wife	None	13	INH/RFP/SM
	M	17	Son	None	19	INH/RFP/SM
A9	M	66	Father*	None	0	INH/RFP/EMB/SM/KM
	F	36	Daughter	None	14	INH/RFP/EMB/SM
A10	M	52	Husband*	None	0	INH/RFP/SM
	F	56	Wife	None	17	INH/RFP/SM
A11	M	60	Father*	None	0	INH/RFP/EMB/SMO
	M	25	Son	None	19	INH/RFP/SM
A12	M	45	Father*	None	0	INH/RFP/SM
	M	23	Son	None	40	INH/RFP/SM
A13	M	15	Son*	None	0	INH/RFP/SM
	M	58	Father	None	40	INH/RFP/SM/OFX
A14	M	37	Son*	None	0	INH/RFP/EMB/SM
	F	67	Mother	T2DM	41	INH/RFP/EMB/SM
A15	M	19	Son*	None	0	INH/RFP/SMK
	F	47	Mother	None	42	INH/RFP/SM
A16	F	48	Mother*	None	0	INH/RFP/SM
	F	25	Daughter	None	43	INH/RFP/SM
A17	M	51	Husband*	None	0	INH/RFP/SM/OFX
	F	53	Wife	T2DM	45	INH/RFP/SM/OFX
A18	F	31	Daughter*	None	0	INH/RFP/OFX
	M	67	Father	None	47	INH/RFP/SM/OFX
A19	F	58	Wife*	None	0	INH/RFP/EMB/SM
	M	68	Husband	None	49	INH/RFP/EMB/SM
A20	F	40	Mother*	None	0	INH/RFP/SM
	F	16	Daughter	None	53	INH/RFP/SM
A21	F	52	Mother*	None	0	INH/RFP/SM/OFX
	F	27	Daughter	None	53	INH/RFP/SM
A22	M	21	Younger brother*	None	0	INH/RFP/SM
	M	25	Elder brother	None	59	INH/RFP/SM/OFX
A23	M	60	Father*	T2DM	0	INH/RFP/SM
	M	44	Son	T2DM	110	INH/RFP/EMB/SM/OFX

^a^*M* male, *F* female

^b^An asterisk indicates the index case

^c^T2DM: diabetes mellitus type 2

^d^*INH* isoniazid, *RFP* rifampicin, *EMB* ethambutol, *SM* streptomycin, *KM* kanamycin, *OFX* ofloxacin

### The relationship between index cases and secondary cases

Thirty cases were parent–child relationship (57.7%), twelve cases were husband and wife relationship (23.1%), six cases were sibling relationship (11.5%), and four cases were relative relationship (7.7%) (Table 1).

### Onset time of secondary cases

The time interval between the onset of the secondary case and the index case was 0.5–110 months, of which thirteen cases occurred within two years, accounting for 50.0%; eight cases occurred at an interval of 3–4 years, accounting for 30.8%; four cases occurred at an interval of 5 years, accounting for 15.4%; one case occurred more than nine years apart, accounting for 3.8%.

### The phenotypic susceptibility results of the secondary cases and the index cases

The phenotypic susceptibility results of the secondary cases and the index cases were consistent in thirteen cases, accounting for 50%, and the phenotypic susceptibility results were inconsistent in thirteen cases, accounting for 50%.

### The results of DTM-PCR and 24-loci MIRU-VNTR genotyping

DTM-PCR typing of twenty-two strains of *Mycobacterium tuberculosis* from eleven pairs of families showed that twenty-one strains belonged to Beijing genotype, and only one strain (A12-1) belonged to non-Beijing genotype.

**Fig. 2:**
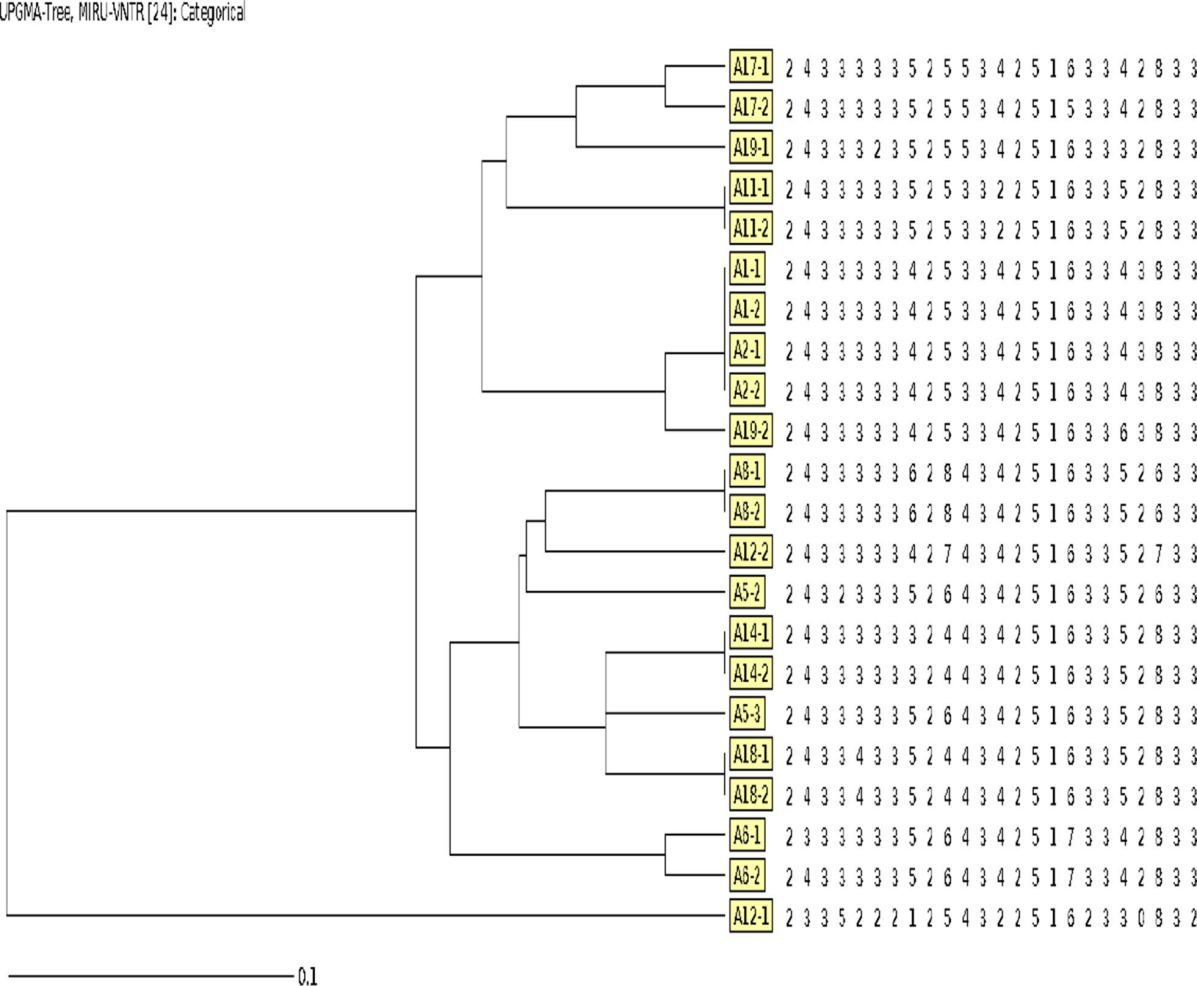
24-loci MIRU-VNTR genotyping results of 22 strains of *Mycobacterium tuberculosis* from 11 families. The 24-digit profile for each strain is displayed in a single row; each number indicates the unit repeats of one locus

We performed 24-loci MIRU-VNTR genotyping on twenty-two strains of *Mycobacterium tuberculosis* from eleven pairs of matched families, and UPMGA method was used to draw the minimum classification tree, as shown in Fig. 2. The genotyping results of six pairs of families were consistent. Three pairs of families (A5, A6, A17) had one locus difference in genotyping results. One pair of families (A19) has four loci differences, and one pair of families (A12) had 12 loci differences.

## Discussion

MDR-TB is a heavy burden on tuberculosis control due to the variety of treatment drugs, long treatment course, high incidence of adverse reactions, and high treatment costs. It may lead to adverse treatment outcomes or deaths to patients, and bring great damage to patients' physical and mental health and their family. Close family contacts are high-risk groups of transmission [13]. A second case or more than one case of MDR-TB in a family will cause great psychological and family financial harm. The proportion of patients with catastrophic family expenditures due to MDR-TB is up to 78% [14]. Follow-up of close contacts of MDR-TB families can identify and treat MDR-TB patients in a timely manner, effectively saving more lives and reducing transmission within the family.

Like ordinary tuberculosis, transmission of MDR-TB is mainly short-range droplet transmission. Because there is no better isolation and protection measures in contact time and space for the family living in the same household, MDR-TB is easier to spread. A previous study reported transmission from close family contacts of smear-positive pulmonary tuberculosis patients in China [15], but few reports were available about the transmission of MDR-TB from close family contacts. Studies from the other countries reported that the incidence of MDR-TB was higher among close family contacts of MDR-TB patients [16, 17].

If there are two or more secondary MDR-TB cases in close family contacts, it is called an outbreak [18]. An outbreak of seven and four MDR-TB cases in close family contacts has been documented Turkey [18] and Taiwan, China [9]. This study collected the cluster incidence of MDR-TB in 23 families and showed that there were three outbreaks of MDR-TB in the three families. This study showed that among the transmission of MDR-TB in family members, the highest transmission rate occurred between parents and children (57.7%), followed by transmission between couples (23.1%), indicating the risk of transmission between parents and children and between couples is high. The risk of transmission between siblings is relatively low, suggesting that MDR-TB transmission is related to close family contact. It has been reported [19] that the risk of onset is the highest within two years of MDR-TB exposure. The results of this study showed that the shortest time interval between the onset of the secondary case and the index case was half a month and the longest interval was 110 months. Fifty percent of the secondary cases had the onset within two years, and 80.8% of the secondary cases had the onset within three years. The results suggested that the follow-up of close contacts of MDR-TB for three years can detect 80% of secondary cases early, then early isolation and treatment can reduce transmission.

Many factors are involved in influencing for the occurrence of MDR-TB. MDR-TB patients can cause great transmission before, during and after the treatment period to potentially harm the surrounding population. Gao Qian et al. [20] believed that transmission is the main cause of high rates of drug resistance to TB in China. A study in Peru [21] showed that 64.3% (27/42) of the secondary cases had the same phenotypic susceptibility results as the index case, and 50% of the secondary cases in this study were consistent with the index case, indicating that there might be intra-family transmission. The phenotypic susceptibility results of 50% of the secondary cases were inconsistent with those of the index cases. There might be two situations: one was that the secondary cases were infected through other routes and were not transmitted within the family; the other was the transmission within families due to the inconsistent stability of drug sensitivity tests for different anti-tuberculosis drugs. INH and RFP have the highest stability [22], while other anti-tuberculosis drugs such as EMB, SM, KM and OFX have poor stability and poor reproducibility, resulting in the same susceptibility results of INH and RFP among family cases, but the drug susceptibility results of other anti-tuberculosis drugs are not consistent.

Diabetes mellites is correlated with increased susceptibility to and disease progression of tuberculosis [23]. Our results showed that six of twenty-six secondary cases had diabetes mellitus type 2 (T2DM), for which the time intervals were 0.5, 10, 46, 41, 45, 110 months respectively. Among them, two cases were diagnosed within one year, four cases were diagnosed after more than three years. This indicated MDR-TB household contacts with diabetes might be susceptible to tuberculosis and should be early screened and followed up for TB infection.

The MIRU-VNTR typing method is a simple and rapid method for *Mycobacterium tuberculosis* genotyping, and also an important tool for TB molecular epidemiology study, which can be used to explore the transmission mechanism of tuberculosis, contact investigation and homology investigation. There were few reports on the genotyping of transmission in MDR-TB families, but most of which were case reports [8, 9]. We used the standard 24-loci MIRU-VNTR genotyping in twenty-two MDR-TB patients, and twelve (54.5%) had exactly the same genotype, and three cases differed in only one locus. If defined as clusters within one locus, intra-family transmission increased to eighteen cases (81.8%), which was basically consistent with the results of family transmission of tuberculosis reported by Augustynowicz-Kopec et al. [24]. As MIRU-VNTR assays discriminate genotypic differences during the transmission process, some loci may be variable during transmission [25]. Cavany SM's genotype among matched tuberculosis close contacts was 79% homologous, which also suggested higher contact transmission [26]. Although there were identical phenotypic drug susceptible test results in two familial strains, the more extensive differences in them precluded intra-family transmission of MDR-TB. Patients from those families could have developed MDR-TB as a result of transmission from outside the household.

The traditional opinion is that bacterial resistance will reduce bacterial fitness, which manifests itself in a lower risk of transmission of resistant organisms [27, 28]. Some studies indicated some resistance mutations reduce growth rates or virulence [29, 30], others argued that some mutations had little or variable impact [31]. Even when mutations do confer fitness costs, subsequent ‘compensatory’ mutations can overcome fitness deficits associated to drug resistance, setting the basis for the spread of DR-TB [32, 33]. Although the transmission of MDR-TB and sensitive tuberculosis are controversial, some authors believed that there was no difference between the transmission of MDR-TB and sensitive tuberculosis at population level [34]. Our results further confirmed that the close family contacts of MDR-TB patients had a higher transmission rate. Personal protection, education, and screening should be carried out in accordance with regulations for close contacts of MDR-TB patients to reduce transmission.

There are several limitations in our study. First of all, in twenty-three matched families, we only collected and genotyped eleven pairs of MTB strains from eleven families, the other samples were not included due to strain loss or death. Second, due to lack of investigation of family contacts of MDR-TB patients, it was impossible to analyze the incidence of MDR-TB in close family contacts. Finally, the lack of investigation in contact networks outside the patient households was a limitation of the current work.

## Conclusions

This study indicates that the family households of MDR-TB patients are at risk for infection of MDR-TB. To reduce transmission, MDR-TB patients should be diagnosed earlier and promptly treated effectively, meanwhile the family contacts should be screened for TB infection and be followed up at least two years.

## Data Availability

The datasets used and/or analyzed during the current study available from the corresponding author on reasonable request.

## Abbreviations

DNA: Deoxyribonucleic acid
DTM-PCR: Deletion-targeted multiplex polymerase chain reaction
EMB: Ethambutol
INH: Isoniazid
IS6110: Insert sequence 6110
KM: Kanamycin
MDR-TB: Multidrug-resistant tuberculosis
MIRU-VNTR: Mycobacterial interspersed repetitive unit variable number tandem repeats
MTB: *Mycobacterium tuberculosis*
OFX: Ofloxacin
RFP: Rifampicin
RR-TB: Rifampicin-resistant tuberculosis
SM: Streptomycin
TB: Tuberculosis
TST: Tuberculin skin test
T2DM: Diabetes mellitus type 2
UPGMA: The unweighted pair-group method with arithmetic mean
WHO: World Health Organization
